# Supplementary material for: Extracellular Vesicle-Packaged miR-195-5p Sensitizes Melanoma to Targeted Therapy with Kinase Inhibitors
Source: Cells. 2023 May 5;12(9):1317. doi: 10.3390/cells12091317 (PMC10177607; doi:10.3390/cells12091317)
Supplement: Supplementary file 1 [file cells-12-01317-s001.zip › cells-2329821-supplementary.pdf]

Supplementary information

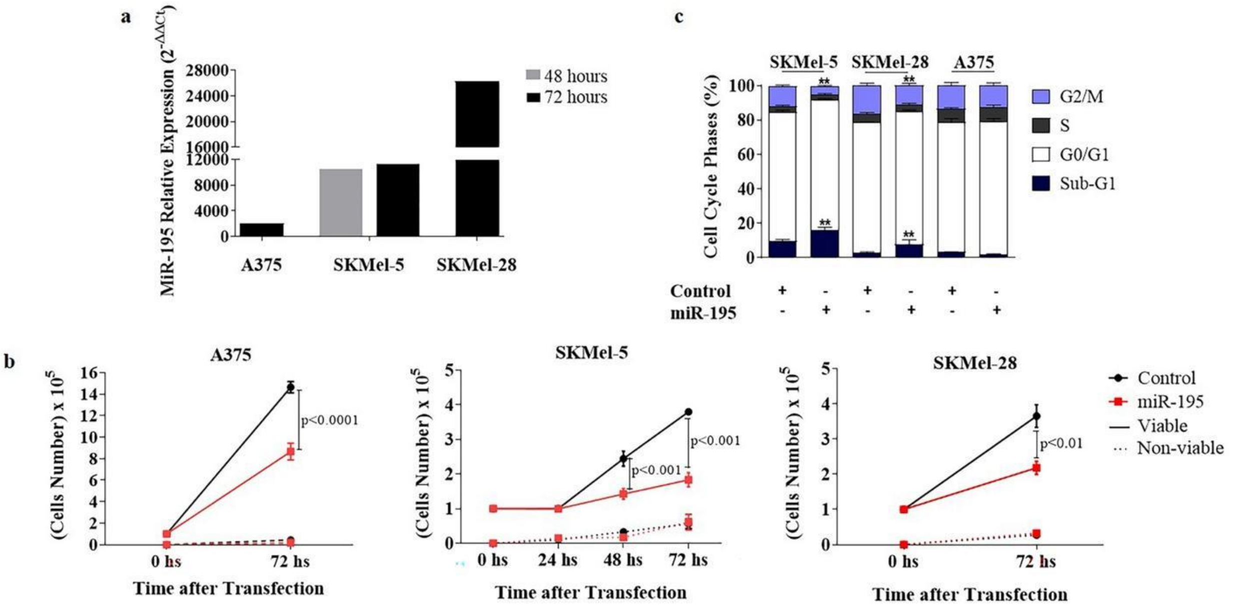

Figure S1. MiR-195-5p overexpression induces cytostatic and cytotoxic effect in human melanoma cells. **a.** miR-195-5p relative expression 48 and 72 h after transfection, compared to scramble cells. RNU48 was used as endogenous control. **b.** Cell number obtained by trypan blue count (n = 5). **c.** Cell cycle profile of propidium iodide (PI)-labeled cells (n = 5).

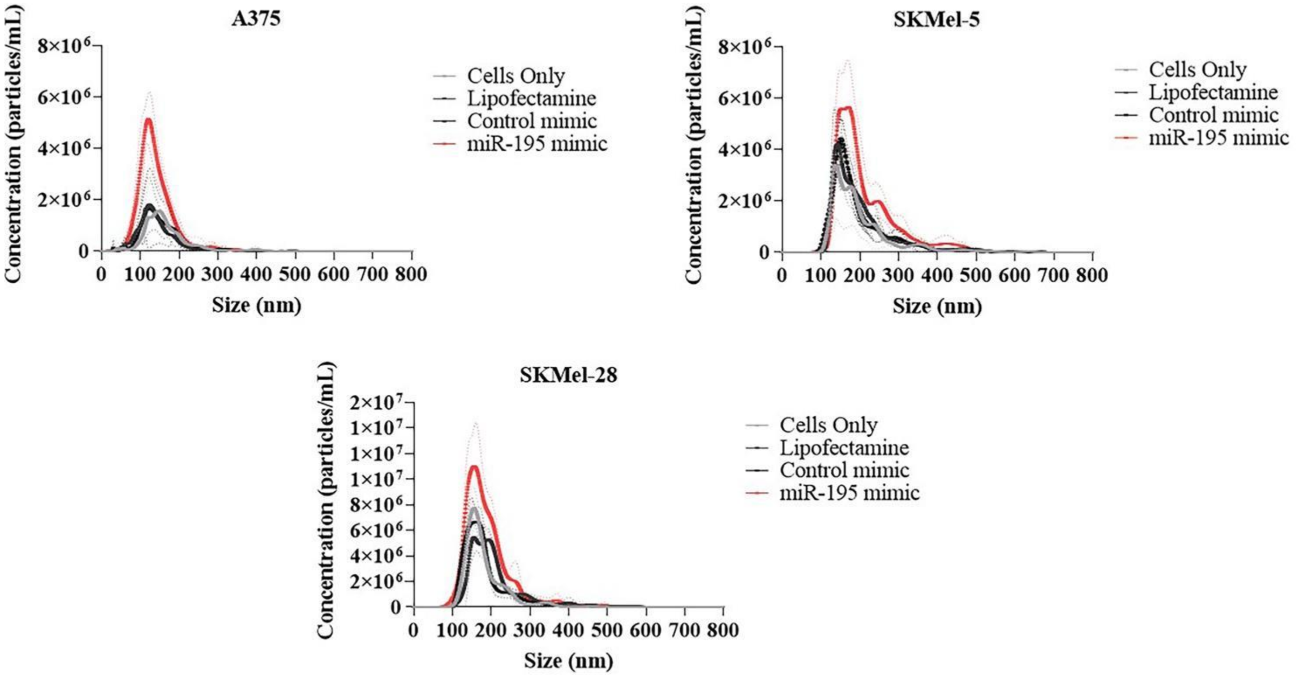

Figure S2. Vesiculation profile of control, lipofectamine and transfected cells confirming that induction of small EVs release is associated with miR-195-5p transfection.

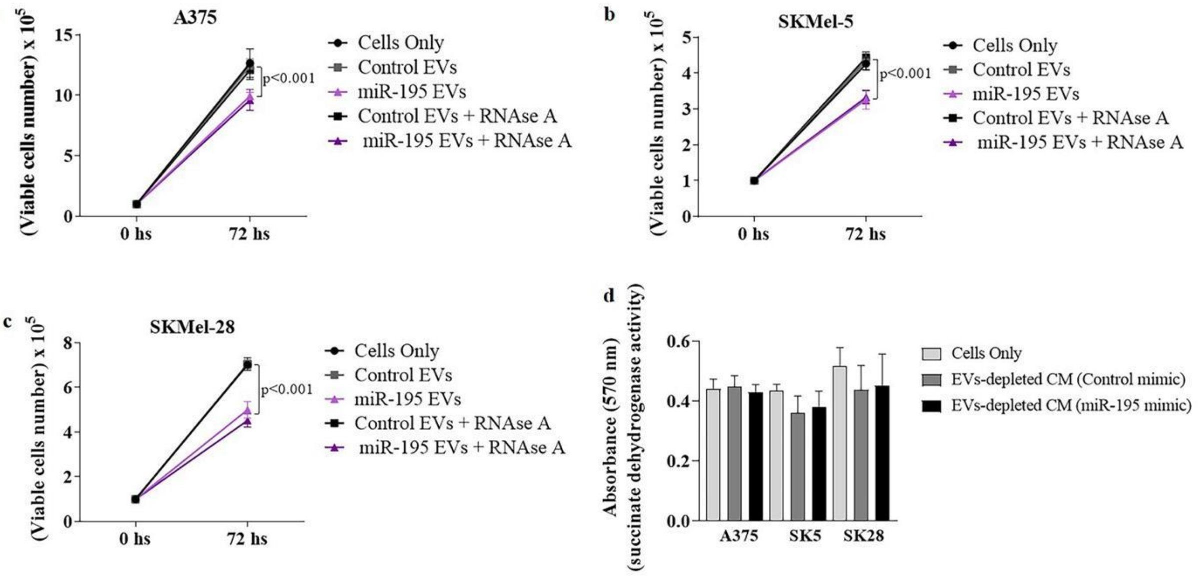

Figure S3. Cytostatic effect in *naïve* cells is exerted through EVs-mediated cargo transfer. **a-c.** Number of viable cells obtained by trypan blue count after EVs treatment with RNAse A (n = 3). **d:** Cell viability, measured by MTT assay, after incubation with EVs-depleted medium for 72 h (n = 8).

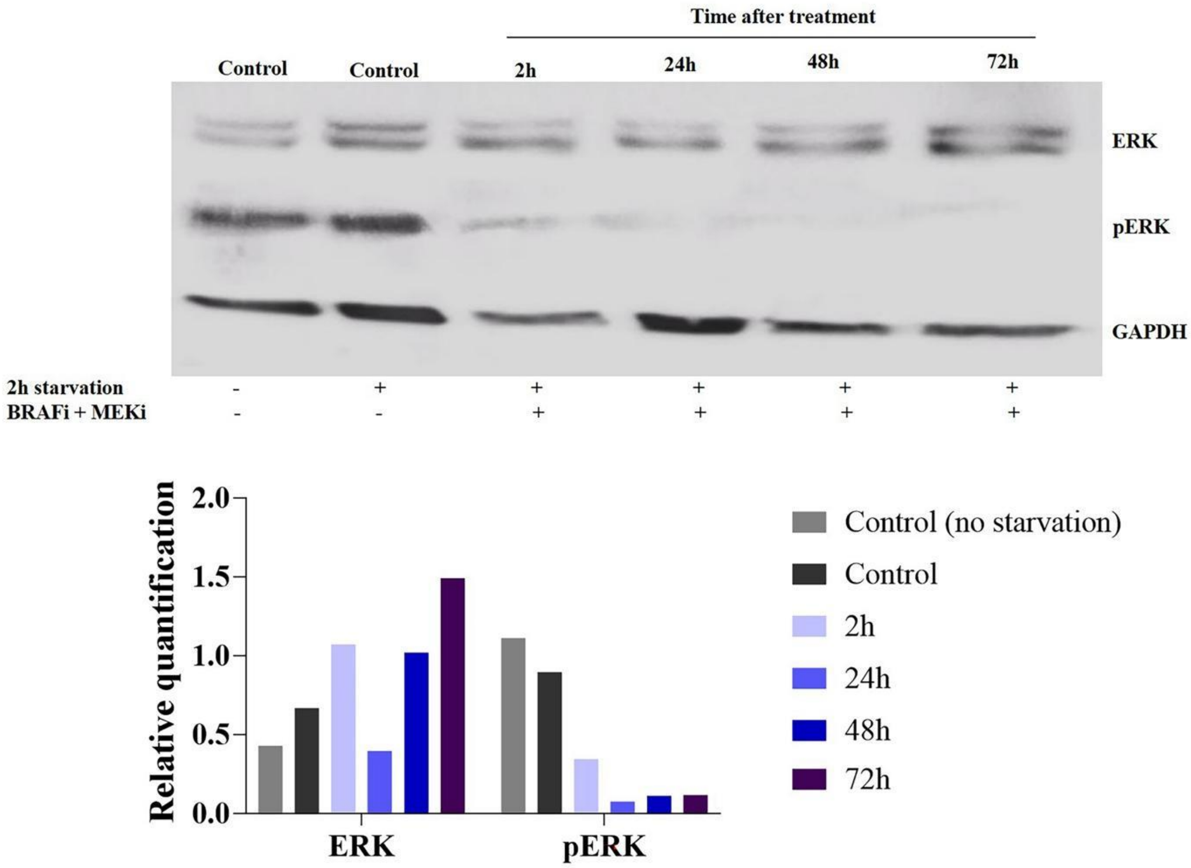

.

Figure S4. Inhibition of ERK phosphorylation after MAPKi treatment.

Western blot showing protein levels of total ERK and phospho-ERK after MAPKi treatment. Cells were previously starved for 2 h (cultured without FBS).

Table S1. Primers sequences used for RT-qPCR.

Gene	Forward	Reverse
**β-*ACTINA***	5′-AGAAAATCTGGCACCACA-3′	5′-AGAGGCGTACAGGGATAGCA-3′
***BCL2*-L1**	5′-ACAGCAGCAGTTTGGATGC-3′	5′-GGTGATGTGGAGCCTGGGATG-3′
CDC42	5' - GCCCGTGACCTGAAGGCTGTCA - 3'	5' - TGCTTTTAGTATGATGCCGACACCA - 3'
GAPDH	5′-TGCACACCAACTGCTTAGC-3′	5′-GGTGGACTGTGGTCATGAG-3′
HPRT	5′-CCCTGGCGTCGTGATTAGT-3′	5′-TCTCGAGCAAGAGGTTCAGT-3′
RAB27A	5' - AGAGGAGGAAGCCATAGCAC - 3'	5' - CATGACCATTTGATCGCACCAC- 3'
RAB27B	5' - GGAACTGGCTGACAAATATGG - 3'	5' - CAGTATCAGGGATTTGTGTCTT- 3'
RAB31	5' - ATCTTTGGGCTGGGTTTG - 3'	5' - ATGGGCTCATTAGTGGGTAG - 3'

Table S2. List of genes analyzed by microfluidic RT-qPCR.

ABCB1	***COL6A2***	***MMP12***	***TGFB1***
ABCC1	*CXCR4*	*MMP9*	*TRAF6*
ACTB	*E2F1*	*MYC*	*TWIST1*
ACVR1B	*EGFR*	*NANOG*	*TWIST2*
AKT1	*EGR1*	*NFE2L2*	*VEGFA*
ALDH1A1	*EIF2AK3*	*NFE2L3*	*VIM*
ATF4	*EPHA2*	*NODAL*	*WNT3A*
ATF6	*ERN1*	*NOTCH1*	*WNT5A*
ATM	*FGF13*	*NOTCH4*	*YAP1*
AXIN2	*FGF2*	*PDCD1*	*YWHAZ*
B2M	*FGF2R*	*PHB*	*ZEB1*
BAD	*FLOT2*	*POU5F1*	*ZEB2*
BAX	*FOS*	*PTEN*	
BBC3	*GAPDH*	*RB1*	
BCL2	*GUSB*	*RHOA*	
BCL2L1	*HIF1A*	*RIPK2*	
BIRC5	*HMGB1*	*RIPK3*	
CASP8	*HPRT1*	*RPLP0*	
CCND1	*IGF1R*	*SNAI1*	
CD274	*JUN*	*SOX2*	
CD44	*KRAS*	*SOX7*	
CDH1	*LAMC2*	*STAT1*	
CDH2	*LATS1*	*STAT3*	
CDH5	*LATS2*	*STK3*	
CDKN1A	*MCL1*	*STK4*	
CDKN2A	*MDM2*	*TAZ*	
CDKN3	*MITF*	*TBK1*	
CFLAR	*MMP1*	*TFRC*	

Table S3. Antibodies used for WB.

Antibody	Dilution (titer)	Brand
**Anti-Calnexin**	1:500	Abcam – ab58504
**Anti-CD63**	1:700	Thermo Fischer Scientific - PA592370
**Anti-CD9**	1:500	Thermo Fischer Scientific - PA5-85955
**Anti-BCL2-L1**	1:1000	Thermo Fischer Scientific - MA5-15142
**Anti-gERK1/2**	1:10000	Sigma – M5670
**Anti-pERK1/2**	1:2000	Sigma – M8159
**Anti-ACTIN**	1:2000	Sigma – A5060
**Anti-GAPDH**	1:1000	Sigma – G8795
**Anti-rabbit IgG Peroxidase**	1:7000	Sigma – A9169
**Anti-mouse IgM Peroxidase**	1:4000	Sigma – M-6274

Table S4. Predicted targets and biding sites.

Gene	Predicted pairing
BCL2-L1 (946-952)<br>miR-202-3p	5'…CCCCAGGGUCUUCCCUACCUCAG…3'<br>3' AAGGGUACGGGAUAUGGAGA 5'
BCL2-L1 (2529-2536)<br>miR-195-5p	5'…GAAUAUCCAAUCCUGUGCUGCUA…3'<br>3' CGGUUAUAAAGACACGACGAU 5'
YAP1 (162-168)<br>miR-195-5p	5'...CUCUUCCUUGUCCAUUGCUGCUG…3'<br>3' CGGUUAUAAAGACACGACGAU 5'
VEGFA (292-299)<br>miR-195-5p	5'...CCAUUUUAUUUUUCUUGCUGCUA…3'<br>3' CGGUUAUAAAGACACGACGAU 5'
CCND1 (1961-1967)<br>miR-195-5p	5'...CCAUUUUCUUAUUGC---GCUGCUAC…3'<br>3' CGGUUAUAAAGACACGACGAU 5'
CCND1 (2033-2040)<br>miR-195-5p	5' ...CUCUUUCACAUUGUU-UGCUGCUA…3'<br>3' CGGUUAUAAAGACACGACGAU 5'
CCND1 (2213-2220)<br>miR-202-3p	5' ...GGUUGCUGUUUCACAAUACCUCA..3'<br>3' AAGGGUACGGGAUAUGGAGA 5'
CCND1 (2873-2879)<br>miR-202-3p	5'...AACACGGCUCACGCUUACCUCAA…3'<br>3' AAGGGUACGGGAUAUGGAGA 5'
CCND1 (2730-2750)<br>miR-152-3p	5'...CCGCACGATTTCATTGAACA…3'<br>3' UCAGUGCAUGACAGAACUUGG 5'
